# An investigation into the robustness of a double-ended wideband impedance-based fault location technique

**DOI:** 10.1038/s41598-023-36541-2

**Published:** 2023-06-20

**Authors:** Hayder K. Jahanger, David W. P. Thomas, Mark Sumner

**Affiliations:** 1 AHIVE Research Centre, Cardiff University, Cardiff, CF24 3AA UK; 2 Power Electronic, Machine and Control (PEMC) Research Group, The University of Nottingham, Nottingham, NG7 2RD UK

**Keywords:** Electrical and electronic engineering, Renewable energy

## Abstract

The double-ended impedance-based fault location technique (DEFLT) uses the wideband frequency content of the transient generated by the fault to determine the impedance from the point of measurement to the fault. This paper evaluates and develops the DEFLT experimentally for a Shipboard Power System (SPS) to determine its robustness to source impedance, the presence of interconnected loads (“tapped” loads) and tapped lines. Results demonstrate that the estimated impedance (and therefore distance to the fault) is influenced by the presence of tapped loads when the source impedance is large, or when the tapped load is comparable to the rated load of the system. Therefore, a scheme is proposed that compensates for any tapped load without requiring any additional measurements. Using the proposed scheme, the maximum error is significantly reduced from 92 to 13%. Simulation and experimental results show that a high accuracy for the estimated fault location can be achieved.

## Introduction

Shipboard Power Systems (SPS) play a vital role in the next generation of naval vessels which will employ more electrical loads for example for propulsion^1^. Fast and accurate fault location are required for SPS to minimize the disruption of power delivery to essential loads and to enhance the reliability and robustness of the system^1–6^. Radial based power distribution is the traditional structure of the SPS. However, Zonal Electrical Distribution (ZED) architectures have also been employed to provide higher survivability, efficiency, and reliability in navy_fleets^2^. Faults are typically short circuit between any two or three lines or between any line and the ground, and high impedance and arc fault are also common^1^. However, the design of a cost-effective and accurate fault location method for small scale SPS, while keeping number of measurements to a minimum, is a challenging task.

Three main protection techniques are usually employed in shipboard power systems: overcurrent, distance, and differential^3,7^. The lengths of cables are typically shorter (about 10–200 m long^7^) than in the large distribution networks and therefore the impedances of the cables are small (about 0.04 Ω/1000 feet^7^). Hence, distance protection in short length power systems is impractical because the impedances of the cables are too low to detect with only a small error. Active Impedance Estimation (AIE) was proposed for fault locations in integrated and shipboard power systems with short cables as an improved distance scheme. A short current pulse is injected and the resulted current and voltage transients are used to estimate the impedance at higher frequencies^8–12^. Analysis at higher frequency increases the cable reactance and simplifies the fault location procedure. Single or multiple injections can be performed depending on the system layout and the protection requirements^8,10–12^. A pulse per phase is injected into the three phases AC SPS^9^, and the distance is calculated by comparing the estimated reactance at higher frequencies to the calibrated cable reactance at the same frequencies. Although, these techniques offer a high accuracy and are “single ended” (i.e. only make measurements from one point in the system), they require additional hardware and cost. The researchers in^13^ proposed voltage injection at a higher frequency (1–7) kHz at different points within the system. The method compares the different measurements to decide which phase is faulty then applies iteration to narrow down the location. Conventional fault detection and location techniques based on symmetrical component were investigated by the authors in^14^ on a radial AC marine system. The authors concluded that the conventional overcurrent approach needs to be improved. Symmetrical components are only useful for detecting faults in the system. On the other hand, differential protection methods can work properly in detecting faults in a SPS with short cables^15^. However, to achieve accurate fault location using the differential approach, a relay is required in each piece of equipment in the SPS and an effective communication system should be provided between the zone and the equipment in order to cover the whole SPS^15^. This technique is more vulnerable to communication system failure and is also not cost-effective^5,8^ due to the use of a relay in each piece of equipment with communication channel.

Another widely researched non-conventional technique for fault location in SPS is Time or Frequency Domain Refectory (TDR or FDR) which is based on the travelling fault transient wave^16–18^. A combination Time–Frequency Domain (TFDR) was proposed in^16^. TFDR is used to estimate the location of the fault by tracking a specific feature in the entire reflected signal. A matching method was combined with TFDR analysis to find the fault location and overcome some of TFDR limitations such as multiple reflections and noise^16^. An approach was proposed in^17^ based on using a forward model to create TDR responses and an inverse optimization technique with the aim of minimizing the difference between the created (simulated) and the measured TDRs. The fault is located by comparing the calculated length using TDR with the healthy branch. The authors investigated the method using a very short and small-scale circuit with a maximum length of 5 m^17^. TDR and FDR however have limitations that affect their accuracy such as the rise time and frequency sweep bandwidth, and both approaches are vulnerable to system noise^16^. Additionally, multiple reflections can make it harder to estimate the fault location and long cables tend to attenuate the TDR pulse heavily^18^.

The first part of this review summarized the active impedance-based fault location techniques, however, most of these techniques requires two injection and a measurement unit. Some of these techniques were tested only with DC SPS and require extra hardware to be installed or embedded in the form of power electronic equipment to generate injection pulses. For the second part, TDR or FDR techniques were presented and these techniques were proposed to work for shipboard systems will attenuate the travelling pulse and adversely affect the location accuracy. None of these techniques addressed the problem of tapped or multi-lateral power systems where load connections between the measurement points could influence the fault transients seen. This paper presents a detailed investigation of a wideband impedance based double-ended fault-location technique (DEFLT) that is particularly developed for a shipboard power system and directly uses the generated fault transients instead of a deliberate signal injection. This technique is simple to perform in a real-time process. The time required to locate the fault could be decreased to as low as 5 ms after the occurrence of the fault with an accuracy of 2 m. The rest of the paper is divided into four sections. "Algorithm review" section describes the proposed algorithm, "Experimental system" section presents the experimental system setup and calibration, "Demonstration through simulation" section presents the simulation evaluation.

## Algorithm review

### DEFLT

**Figure 1:**
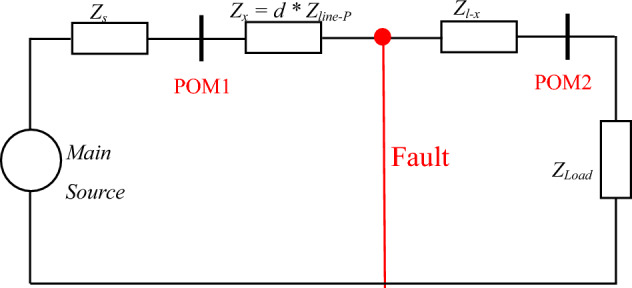
Single phase system with a short circuit fault.

**Figure 2:**
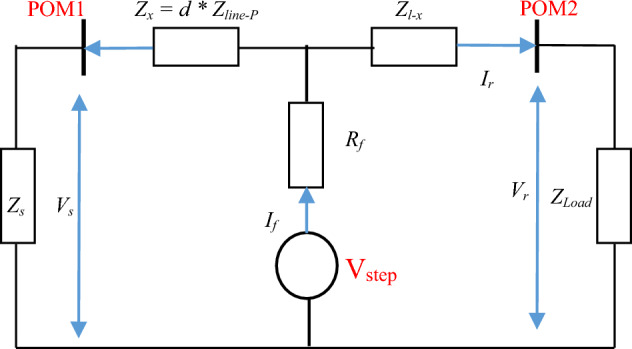
System at non-fundamental frequency during fault situation.

A simple single-phase system shown in Fig. 1, where *Z*_*s*_ is the source impedance, *Z*_*load*_ is the load impedance. The impedance between the fault and the source (the sending end) is *Z*_*x*_ and the remaining impedance *Z*_*l-x*_ represents the impedance to the load (receiving end). It is known that a low resistance or a short circuit fault causes a step voltage transient at the fault location and this contains information over a wide frequency range. This fault transient can be considered as a voltage source V_step_ at non-fundamental frequencies as shown in Fig. 2. where POM1 and POM2 are the measurements points at the sending and receiving ends respectively. The impedance between POM1 and the fault point can be determined as follows by calculating the fault voltage^19–21^:

1 $$V_{s} + I_{s}Z_{x} = V_{r} + I_{r}Z_{l - x}$$

*V*_s_, *I*_s_ and *V*_r_, *I*_r_ are the measured voltage and current at the sending (s) and receiving (r) ends, *I*_*f*_* and R*_*f*_ are the fault current and fault resistance. The total line impedance is *Z*_*l*_ = *Z*_*x*_ + *Z*_*l−x*_, rearrange (1) yield

2 $$Z_{x} = \frac{V_{r} - V_{s} + I_{r}Z_{l - x}}{{I_{s} + I_{r}}}$$

3 $$d = \frac{imag(Z_{x})}{{imag(Z_{line-p})}}$$

The impedance between the fault point and source end is estimated using (2). Note that (2) is usually calculated in the frequency domain, calculating (2) for each frequency considered. The fault location can be found by dividing the estimated reactance part of ${\mathrm{Z}}_{\mathrm{x}}$ *Z*_*x*_ by the reactance part of the known per-unit length impedance of the line (*Z*_*line-p*_) using (3) at each frequency in the range 250–3000 Hz, and then finding the average of these values. Resistance is neglected as reactance dominate at higher frequencies and to minimise the impact of the fault resistance. The DEFLT does not require the knowledge of the load or the supply impedance as they do not appear in (2).

### DEFLT with a fault on a tapped line

**Figure 3:**
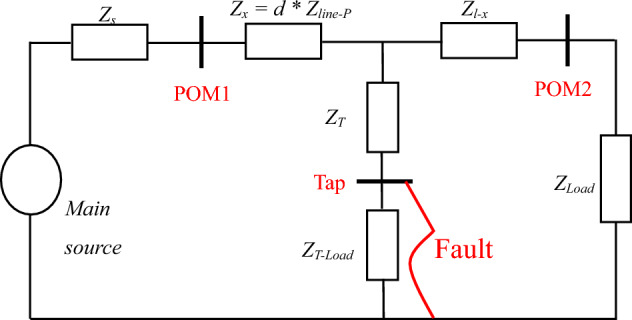
System with fault on tapped-line.

**Figure 4:**
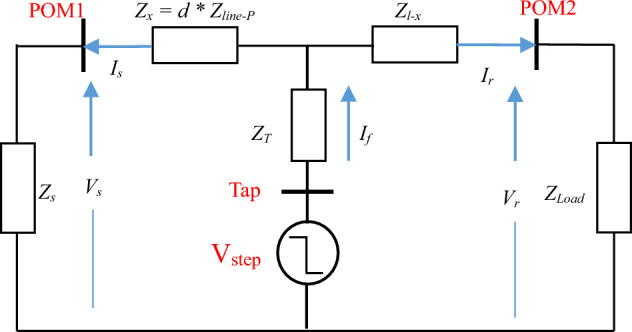
System at non-fundamental frequency during fault on Tapped line using double-ends measurement.

This system is modified to include a tapped line between the measured terminals as shown in Fig. 3, with the equivalent system at non-fundamental frequencies shown in Fig. 4. If a fault occurs on this tapped line, the load on the tapped line (*Z*_*T-load*_) is assumed to be short-circuited by the fault (and therefore not included in the analysis), by calculating the voltage at the tapping point P it can be shown that (1)–(3) are still valid, but the distance estimated is now the distance to the tapping point: the tapped line impedance (*Z*_*T*_) is considered as part of the fault impedance. Consequently, the DEFLT is unable to locate faults on the tapped line. Nonetheless, it has the ability to locate the faulted tapped line which is useful information for the system operator.

### The DEFLT with tapped load compensation

**Figure 5:**
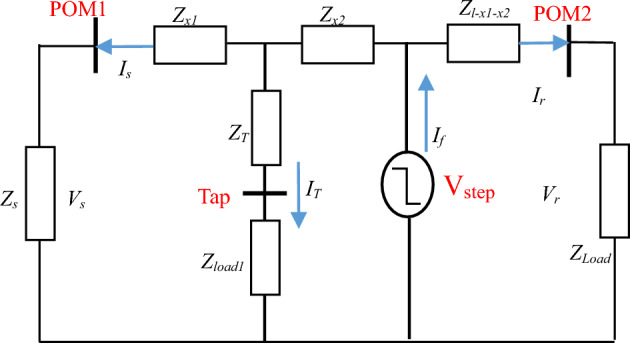
System at non-fundamental frequencies during a fault between the tapped line and POM2 using DEFLT.

This section proposes an extension to the DEFLT which compensates for tapped loads connected between the sending and receiving ends of the line by updating the estimation algorithm based on Fig. 5. Defining *Z*_*x2*_ as the impedance between the Tapped load and the fault:

4 $$Z_{x} = Z_{x1} + Z_{x2} = \frac{V_{r} - V_{s} + I_{r}Z_{l} - I_{T}Z_{x2}}{{I_{s} + I_{r}}}$$

*I*_*T*_ is the tapped-load current, which is not known, and can be estimated assuming the voltage across the tapped load is equal to the source voltage as follows:

5 $$I_{T} = \frac{V_{s} + (I_{s}Z_{x1})}{{Z_{T} + Z_{load1}}}$$

**Figure 6:**
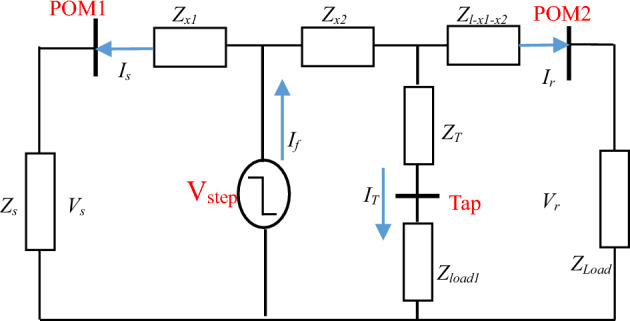
System at non-fundamental frequencies during a fault between the tapped line and POM1 using DEFLT.

If the tapped load is between the fault and POM2 as shown in Fig. 6, the estimation equation is modified as follows:

6 $$Z_{x1} = \frac{V_{r} - V_{s} + I_{r}Z_{l }+ I_{T}Z_{x2}}{{I_{s} + I_{r}}}$$

7 $$IT = \frac{V_{r} + (I_{r}Z_{x3})}{{Z_{T} + Z_{load1}}}$$

where *Z*_*x3*_ is *Z*_*l-x1-x2*_*.* Further detail of derivation of (4) to (7) is shown in Appendix 1.

This new technique (the DEFLT with tapped load compensation) requires only the knowledge of the tapped load position, while the impedance of the tapped line and its load can be estimated. It can then be processed as follows:

1. Calculate an initial estimate of the distance (d0) based on the non-compensated DEFLT (3) in order to locate the fault with respect to the tapped line position.
2. Using voltage and current measurements made just before the fault occurs (pre-fault), the total load seen from the sending end is estimated as ($Z_{total} = \frac{V_{s}(f)}{{I_{s}(f)}}$) while the receiving end load is estimated from the receiving end measurements as ($Z_{load} = \frac{V_{r}(f)}{{I_{r}(f)}}$). The tapped line and its load are then approximated by *ZT* = (*Z*_*total*_ ∗ *Z*_*load*_/*Z*_*load*_ − *Z*_*total*_) assuming *Z*_*load*_ in parallel with *Z*_*T*_.
3. Using knowledge of the location of the tapped line to select a compensation technique.
  - For a fault after the tapped line, *I*_*T*_ is estimated based on the calculated distance using (5).
  - For a fault before the tapped line, *I*_*T*_ is estimated based on the calculated distance using (7).
4. Calculate *Z*_*x2*_ = *Z*_*x*_ − *Z*_*x1*_ based on the distance calculated in the previous iteration (dk-1).
5. Calculate *I*_T_*Z_*x2*_.
6. Calculate new *Z*_*x*_ (impedance between the POM1 and the fault).
7. Determine the reactance of *Z*_*x*_ and divide this by the per-meter reactance of the line at the frequencies (250–3000) Hz and then average the result in order to find the new estimate d_k_. The chosen frequency range offers better SNR and lower aliasing effect.
8. Repeat steps 3 to 7 until the distance estimate converges to within a pre-set tolerance (d_k_ − d_k-1_ < 0.5 m).

## Experimental system

**Figure 7:**
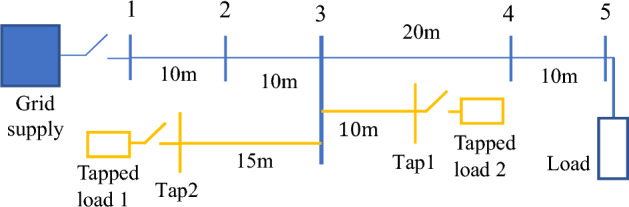
Single line diagram of the experimental setup.

**Figure 8:**
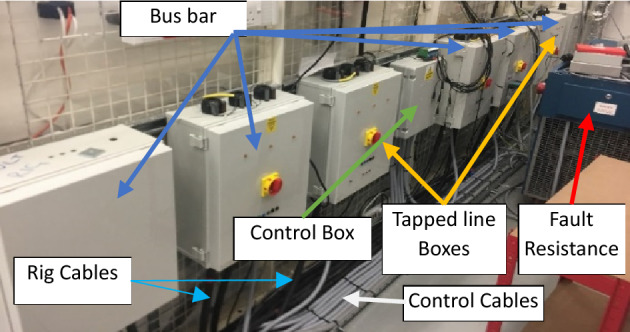
Actual experimental circuit setup.

A radial experimental network similar to a small scale SPS has been constructed to validate the modifications to the DEFLT proposed in this paper. The circuit consists of a 16 mm, 5 core distribution (99A) cable as well as two tapped-line cables with the same cross-sectional area. Different resistive loads can be connected to the receiving end and the ends of the tapped lines as shown in the diagram of Fig. 7. The laboratory setup is shown in Fig. 8. The main cable is subdivided into four sections of which three sections have the same length (10 m) and one section is 20 m long. The two tapped-line cables are connected to the end terminal of "Algorithm review" section (bus 3). The rig is supplied directly from a local 415 V 50 Hz transformer. The 64 Ω per phase load is connected in star to the receiving end (bus 5). Mechanical contactors impose the fault with different fault resistance in any of the five possible locations. The impedance of the cables has been calibrated at chosen frequencies using a Impedance Analysis Interface^22^ as given in Tables 1 and 2. The tables show the small differences in inductance seen between the different cores of the multi-core cable.

**Table 1:** Calibration for brown-blue cores.

Freq. (Hz)		Length			
		10 (m)	20 (m)	40 (m)	50 (m)
500	L (µH)	7.83	14.69	27.2	34.7
	R (mΩ)	28.5	54.2	108.4	142.8
1000	L (µH)	7.7	14.85	26.9	34.0
	R (mΩ)	29.7	25.8	114.9	150.9
1500	L (µH)	7.54	14.6	26.4	33.4
	R (mΩ)	32.7	64	125.2	163.8
2000	L (µH)	7.44	14.4	25.9	32.8
	R (mΩ)	35.36	70.0	136.8	178.3
2500	L (µH)	7.36	14.1	25.4	32.3
	R (mΩ)	38.7	76.4	150	194.4
3000	L (µH)	7.25	13.9	25.07	31.8
	R (mΩ)	42	83.5	162.5	210.7

**Table 2:** Calibration for brown-black cores.

Freq. (Hz)		Length			
		10 (m)	20 (m)	40 (m)	50 (m)
500	L (µH)	10.5	20.5	37.3	46.9
	R (mΩ)	29.0	55.3	106.1	132.9
1000	L (µH)	10.33	19.8	36.8	46.3
	R (mΩ)	31.4	62.16	118.0	148.2
1500	L (µH)	10.1	19.34	36.0	45.3
	R (mΩ)	35.6	70.7	134.6	169.1
2000	L (µH)	9.9	19.0	35.2	44.33
	R (mΩ)	40.2	80.0	154.0	192.6
2500	L (µH)	9.7	18.6	34.5	43.3
	R (mΩ)	45.5	90.5	173.6	217.0
3000	L (µH)	9.52	18.3	33.7	42.6
	R (mΩ)	50.5	100.7	194.0	242.7

### Data acquisition and processing

**Figure 9:**
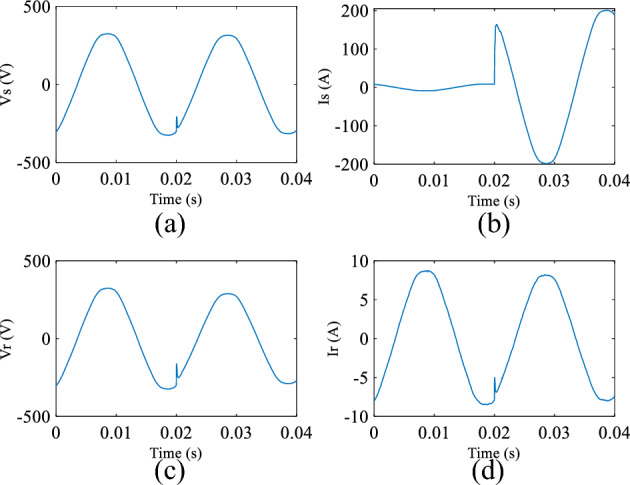
Measured data (**a**) source voltage, (**b**) source current, (**c**) load voltage, (**d**) load current.

**Figure 10:**
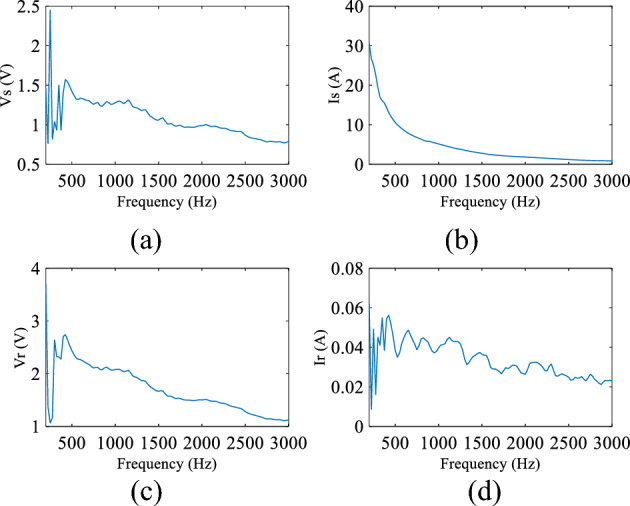
Captured date in Frequency domain (**a**) source voltage, (**b**) source current, (**c**) load voltage, (**d**) load current.

A National Instrument (NI) data acquisition unit has been used to collect measured data from bus 1 and bus 5 and store it on a PC for analysis. The unit consists of two main parts; NI CompactDAQ Four-Slot USB Chassis (NI cQAD-9147)^23^ and the acquisition part (NI 9222)^24^ which captures two sets of voltage and current with a 16 Bit Analog to Digital Converter (ADC) which offers a high resolution. Input signals on each channel are filtered (a 12.5 kHz first order analogue low pass filter is used), buffered, and then sampled by an ADC. A sampling frequency of 200 kHz is used to capture the data because it offers a good SNR for the required frequency range of interest, whilst limiting the sample frequency to a value acceptable for commercial implementation. A sample of the voltage and current measured during a typical fault condition at POM1 and POM2 are shown in Fig. 9. Finally, the signals are converted to the frequency domain using a FFT as shown in Fig. 10 in order to be processed using the appropriate DEFLT.

## Demonstration through simulation

**Figure 11:**
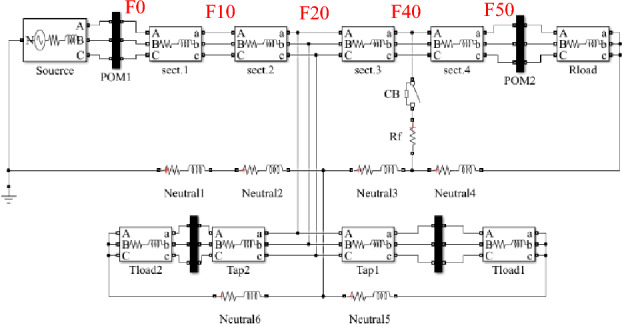
MATLAB Simulation of the experimental system.

**Figure 12:**
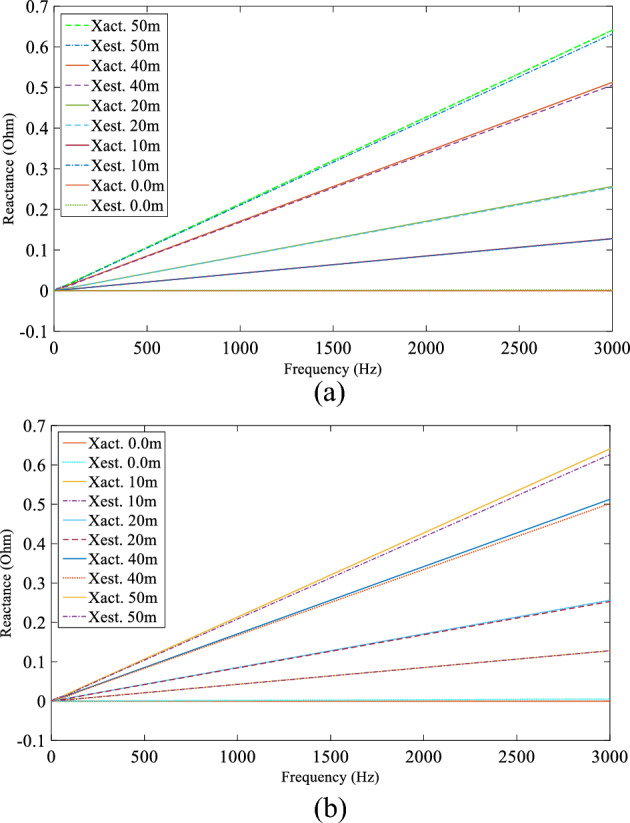
Estimated reactance for L-N fault (**a**) Rf = 1.5 Ω, (**b**) Rf = 4.5 Ω.

**Figure 13:**
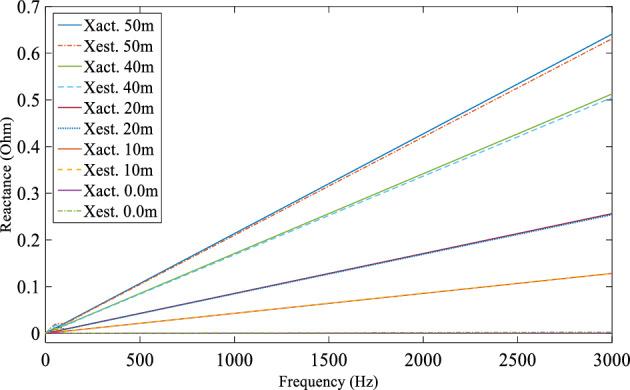
Estimated reactance for Line-Line faults.

**Figure 14:**
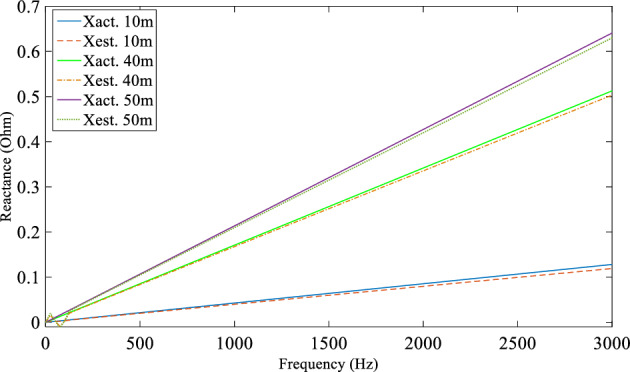
Estimated reactance with a 10 Ω load on tapped line 1.

A computer simulation using MATLAB/Simulink was performed based on the experimental system shown in Fig. 7 in order to demonstrate the DEFLT with the parameters are given in the appendix 2. The simulated circuit is shown in Fig. 11. Five faults are imposed separately as follows; F0 (fault on POM1, F10, F20 (fault on end of Tapped line 1), F40 and F50 (fault on POM2). A Line-Neutral (L-N) fault using two fault resistances 1.45 Ω and 4.5 Ω was imposed and the summary of the estimated reactance versus the actual reactance is presented in Fig. 12. Xact. Means the actual reactance to the fault location while Xest. means the estimated reactance to the fault location. The estimated reactances for the Line-Line (L-L) fault with fault resistance 4.5 Ω are summarised in Fig. 13. The percentage error calculation for the L-N fault presented in Table 3 shows an excellent accuracy. This is because the system has no measurement noise, no data acquisition quantization noise and there is no cable calibration error. It is important to notice that all the F20 reactance estimations presented here are imposed on the terminal of tapped line 1. This verifies that the DEFLT is not able to locate a fault on a tapped line. However, it is able to identify the faulted tapped line. The effect of the size of the loads was also investigated using the simulation study. A 10 Ω star connected load is connected to the terminal of tapped line 1 as shown in Fig. 11 and a 91 Ω load to the terminal of tapped line 2. The estimated reactance using the Simulink measurements are presented in Fig. 14 for F10, F40 and F50. The simulation offers a high accuracy even with the connection of tapped load between the measurement terminals.

**Table 3:** Percentage error for Line-Neutral fault.

Act. fault distance (m)	(R_f_ = 1.45 Ω)		(R_f_ = 4.5 Ω)	
	Est. fault distance (m)	Error (%)	Est. fault distance (m)	Error (%)
0	0.31	0.31	0.85	0.85
10	9.93	− 0.13	9.98	− 0.03
20	19.78	− 0.44	19.73	− 0.5
40	39.45	− 1.2	39.45	− 1.73
50	49.28	− 1.44	48.835	− 2.3

The value of the source impedance (Z_s_) compared to the load impedance plays an important role in the power system as a measure of the system strength. Therefore, the effect of Z_s_ was investigated by increasing its value and keeping the receiving end and tapped loads as 37+j0.5 Ω and 91+j0.4 Ω respectively. Table 4 shows a summary of the estimated distance and resulting percentage error when Z_s_ is increased gradually for a single fault test 40 m from source end, with R_f_ = 1.5 Ω. Increasing the reactance to Z_s_ = 0.051+j0.314 Ω, the error reached 7% of the total main line length. This is because a large source inductance will reduce the size of the fault transients measured at the sending end which reduce the magnitude of the useful high frequency content. This high Z_s_ is tested to protect the cables from high overcurrent during faults which limit the short circuit current to 5 times the rated capacity of the cables.

**Table 4:** Effect of Zs on the DEFLT.

Z_s_ (Ω)	Z_r_ (Ω)	Z_tap_ (Ω)	Act. dist. (m)	Act. dist. (m)	Error (%)
0.00051+j0.0031	37+j0.5	91+j0.4	40	39.15	− 1.7
0.0051+j0.0314				39.35	− 1.3
0.051+j0.0314				39.34	− 1.31
0. 51+j0.0314				39.3	− 1.4
0.051+j0.314				36.65	− 6.65

The second factor to be quantified is the fault resistance. Fixing Z_s_ to 0.51+i0.031 Ω, the receiving end load to 37+j0.5 Ω, and the two tapped loads to 91+j0.9 Ω and 64+j0.7 Ω, an analysis was made using one fault location with different fault resistances. The summary of the system parameters, the estimated distance and the error is presented in Table 5. It is clear that the error increases as R_f_ was increased. This is explained by the fact that the magnitude of the fault generated transient decreases as the fault resistance increases which results in a lower SNR, thus creating a larger estimation error. However, the error only increased by 3% which is not significant compared to Z_f_ was increased from 0.12 to 97% of Z_r_. The last three rows in Table 5 present the estimation when a reactance was added to the fault resistance. The error shows a reduction in value compared to the case when no reactance is included, this is because the reactance damps the transient which filters out some of the unwanted wideband frequency as well as system noise.

**Table 5:** Effect of Z_f_ on the DEFLT with and without noise in the system.

Z_tap2_ = 91+j0.9 Ω, Z_tap1_ = 64+j0.7 Ω, Z_s_ = 0.5+0.0314 Ω			Without NOISE		With 1% noise	
Z_f_ (Ω)	Z_r_ (Ω)	Act. dist. (m)	Est. dist. (m)	Error (%)	Est. dist. (m)	Error (%)
0.12Z_r_	37+j0.5	50	50.18	0.36	49.81	0.377
0.24Z_r_			49.53	− 0.95	49.41	− 1.2
0.48Z_r_			48.95	− 2.01	48.85	− 2.32
0.73Z_r_			48.44	− 3.12	48.4	− 3.2
0.97Z_r_			47.95	− 4.1	48.06	− 3.87
(0.97−0.013i) Zr			48.96	− 2.1	48.86	− 2.28
(0.97−0.005i) Zr			49.9	− 0.33	50.13	0.26
(0.98+0.072i) Zr			50.11	0.22	50.19	0.4

**Figure 15:**
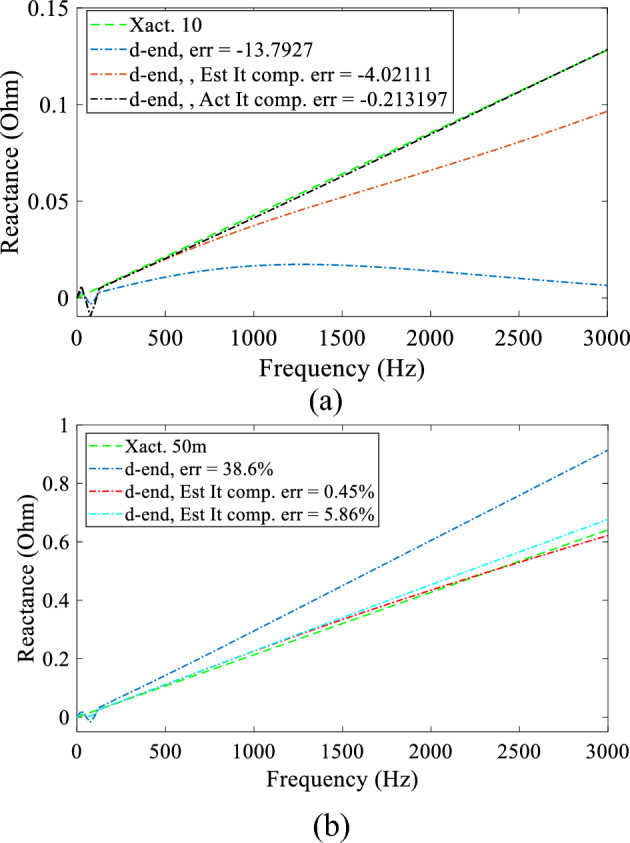
Estimated reactance with 5 Ω equivalent tapped load (**a**) fault 10 m from POM1, (**b**) fault 50 m from POM1.

The effect of tapped loads was then investigated in order to quantify the accuracy of the DEFLT for a more realistic system. Two fault tests were imposed, firstly with a fault between the tapped load point and the receiving end, and then before the tapped load point 10 m from the source end. A sample of the effect of a 10+j0.5 Ω (or 0.27*Z_r_) equivalent tapped load for each fault test is presented in Fig. 15. It is important to mention that the Z_s_ = 0.1+j0.31 Ω and R_f_ = 4.5 Ω. It is obvious that the estimated reactance using (2) has a large error (38.6% and − 13.79%) as plotted with the blue dash-dotted lines (d-end, err =). The effect of the DEFLT with tapped load compensation described in “The DEFLT with tapped load compensation” section can also be seen in Fig. 15, as the red dash-dot line (d-end, Est It comp. err=), and it is clear that it provides a much improved estimation of the fault location with error reduced to − 4% and 0.45% receptively. The DEFLT with estimated tapped load current is compared to an estimation made with the actual tapped load current as plotted with the black dash-dotted lines (d-end, act It comp. err=) and they are obviously very close. This verifies the accuracy of the proposed DEFLT with tapped load compensation.

Finally, a summary of the estimated distances and errors are presented in Table 6 for a fault imposed at 50 m and in Table 7 for a fault imposed at 10 m from POM1. The tapped load impedance (Z_tap_) is reduced from 0.473*Z_r_ to 0.108*Z_r_. It is noticeable that the estimated distance and the error offer an acceptable accuracy when Z_tap_ is 0.473*Z_r_ or larger, whilst the estimated distance and the error begin to diverge largely when the Z_tap_ is comparable to the fault resistance or is smaller.

**Table 6:** Effect of various tapped loads with and without tapped load compensation technique for fault at 50 m.

Z_tap_ (Ω)	Z_r_ (Ω)	Act. dist. (m)	Without tap compensation		With tap compensation	
			Est. dist. (m)	Error (%)	Est. dist. (m)	Error (%)
0.473 * Z_r_	37+j0.5	50	6.69	23.4	53.9	7.8
0.351 * Z_r_			66.0	32.1	54.75	9.5
0.27 * Z_r_			65.94	31.88	48.96	− 2.1
0.1757 * Z_r_			79.3	58.63	50.13	0.27
0.108 * Z_r_			88.47	79.94	44.65	10.7

**Table 7:** Effect of various tapped load with and without tapped load compensation technique for fault at 10 m.

Z_tap_ (Ω)	Z_r_ (Ω)	Act dist (m)	Without tap compensation		With tap compensation	
			Est. dist. (m)	Error (%)	Est. dist. (m)	Error (%)
0.473 * Zr	37+j0.5	10	6.03	− 8.0	8.4	− 3.2
0.351 * Z_r_			4.62	− 10.76	8.03	− 3.95
0.27 * Z_r_			3.1	− 13.8	7.99	− 4.02
0.1757 * Z_r_			0.24	− 19.52	9.2	− 1.59
0.108 * Z_r_			− 2.75	− 25.5	12.95	− 5.9

The error reached 80% for a fault at 50 m or − 25.5% for a fault at 10 m when the Z_tap_ was 0.108*Z_r_. As mentioned earlier, with this small impedance, the tapped load current becomes comparable to the fault current and neglecting it causes a large error. Nevertheless, the proposed DEFLT with tapped load compensation offers a very good compensation for the error caused by the tapped load. The maximum errors are decreased from 80 to 11% without any measurement from the tapped load terminals. The error when using the DEFLT with compensation will be greater than 15% if the source reactance (X_s_) increases above j0.31 Ω, as the fault current will be limited, and the transient is further damped. However, X_s_ of j0.31 Ω is very high and not realistic.

## Experimental results

### Demonstration of DEFLT

**Figure 16:**
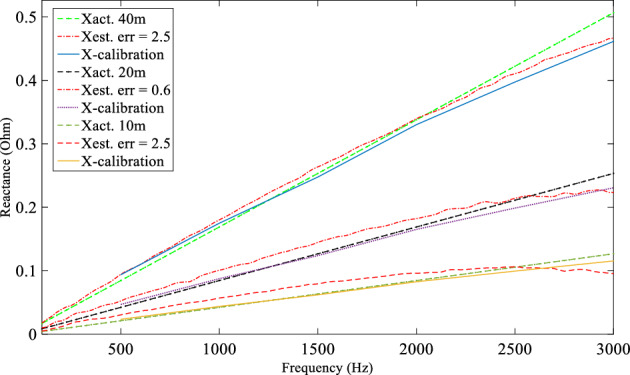
Estimated reactance for three fault tests, R_f_ = 1.45 Ω.

Three tests were performed with a fault resistance of 1.45Ω imposed between line and neutral at F10 (10 m from source end), F20 (20 m from source end on the tapped line) and F40 (40 m from source end) to validate the basic DEFLT. Voltages and currents are measured from Bus 1 and Bus 5 of Fig. 7. The reactance between the Bus 1 and the fault location was calculated using (2). The estimated reactance (red dash dotted lines Xest.) are given in Fig. 16 and compared with actual line reactance (green and black dashed lines, Xact.) and the calibrated reactance (blue solid line, X-calibration from Tables 1 or 2). Note that Xact. uses the inductance at 1 kHz from the X-calibration Tables and assumes it is remains constant over the 50–3000 Hz range. The resistance is neglected because the reactance dominates at higher frequencies: this also removes the effect of the fault resistance from the final estimation. The estimated fault distance for the three tests is summarised in Table 8. The distance is calculated by dividing the estimated reactance over the actual per meter reactance at each frequency and then taking the average of the estimated distances at the selected range. The results presented show a good accuracy—the largest error in distance is 1.25 m.

**Table 8:** Estimated distance and percentage error.

Act. fault distance (m)	(R_f_ = 1.45 Ω)		(R_f_ = 4.5 Ω)	
	Est. fault distance (m)	Error (%)	Est. fault distance (m)	Error (%)
10	9.36	− 1.28	9	− 2.0
20	20.3	0.6	19.23	− 1.5
40	38.75	− 2.5	38.0	− 4.0
50	50.1	0.2	48.05	− 3.9

**Figure 17:**
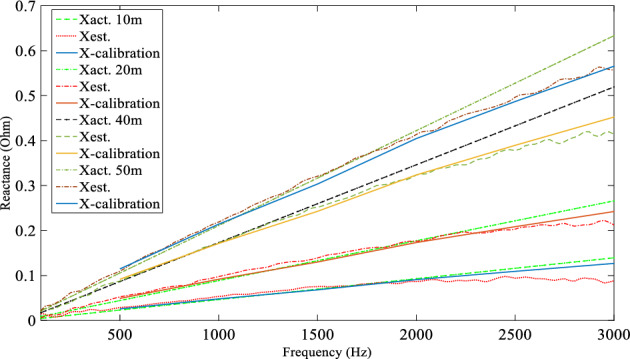
Estimated reactance for 4 fault tests, Rf = 4.5 Ω.

**Figure 18:**
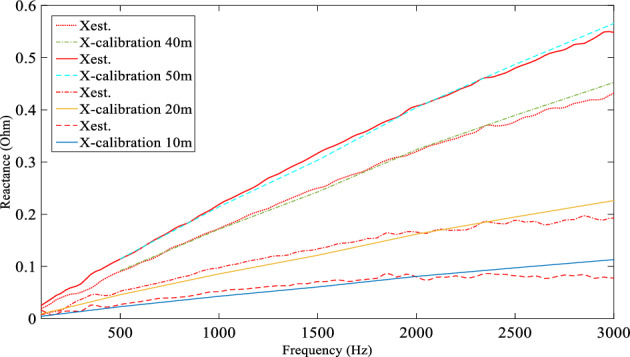
Estimated reactance for L-L fault in different locations.

The same test procedure is repeated but with a higher fault resistance (R_f_ = 4.5 Ω) in order to demonstrate the DEFLT operation for different fault resistances. The estimated reactance using (2) is summarised in Fig. 17. Compared with the calibrated reactance, the estimated reactance shows good accuracy. It important to point out that the F20 test is actually imposed on the tapped line of the system of Fig. 7. The estimated reactance showed that the fault is located 20 m from source-end. The DEFLT considers the tapped line as part of the fault. The estimated distance and the calculated percentage error of the total line length for L-N fault R_f_ = 4.5 Ω are summarised in Table 8. The error increases as Rf increases. This is due to a weaker generated fault transient as Rf increases, resulting in a lower SNR for the measured data. However, the maximum error is 4% which still within the acceptable range of distance error of 2 m. Finally, the DEFLT is further tested with a Line-Line (L-L) fault. The R_f_ used for this test is 4.5 Ω in order to limit the fault current within the limitation of the current transducer used in the experimental rig. The estimated reactances for tests made at all the fault locations are summarised in Fig. 18. The results show a high accuracy compared to the calibrated reactance as summarised in Table 9. The largest error in estimated fault distance is 2 m with estimated distance of 38 m for F40. The L-L fault results showed a slightly better accuracy compared to L-N faults because the L-L fault generates a larger transient with a better SNR.

**Table 9:** Percentage error calculation.

Act. fault distance (m)	R_tap_ = 0.0 Ω		R_tap_ = 10 Ω	
	Est. fault distance (m)	Error (%)	Est. fault distance (m)	Error (%)
10	9.0	− 2	8.9	− 2.2
40	38.0	− 4	38.0	− 4.0
50	48.05	− 3.9	48.0	− 4.0

### DEFLT with tapped load compensation

**Figure 19:**
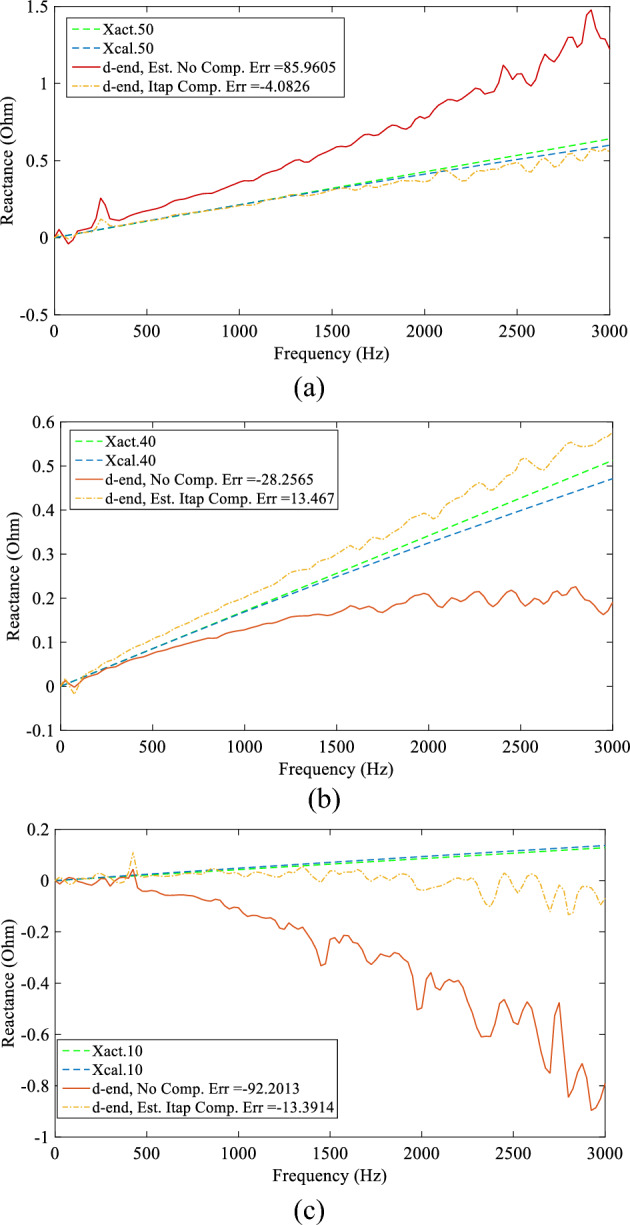
Estimated reactance with and without tapped load compensation (**a**) F50, (**b**) F40, (**c**) F10.

A 10 Ω resistor is used as a load at the Tap1 terminal and a 91 Ω is placed at the Tap2 terminal in addition to the 37 Ω main load on the receiving end terminal (Bus 5). The source inductance is 1mH. Three faults were then imposed on the main line with R_f_ = 4.5 Ω for testing the DEFLT in the presence of tapped loads between the measurement points. The estimated reactances with and without tapped load compensation are presented in Fig. 19. The red solid lines present the estimation without compensating for tapped loads, while the yellow dash-dotted lines present the estimate with the tap compensation method. These are compared with the calibrated reactance given by the blue dashed line and the actual reactance (green dashed line). It is obvious that the tapped load has a significant adverse effect when the source impedance is high, and the magnitude of the tapped load is close to the receiving end load. The error increased to 92% when the combined tapped load was 9 Ω. A summary of the distance and the percentage error are presented in Table 10. It is clear that the DEFLT with tapped load compensation reduced the error in estimation significantly without any measurements from the tapped load. The DEFLT with compensation has demonstrated a potential enhancement in the estimated accuracy by reducing the maximum error to less than 15% without any measurement from the tapped loads.

**Table 10:** DEFLT with tapped load compensation.

Act. fault distance (m)	No compensation		With compensation	
	Est. fault distance (m)	Error (%)	Est. fault distance (m)	Error (%)
10	− 36	− 92	3.3	− 13.4
40	31.2	− 28.2	45.67	13.67
50	92.5	− 85	48.0	− 4.08

## Conclusion

The DEFLT that uses the fault generated transient was investigated in this work using both a MATLAB simulation and an experimental representation of a simple Shipboard Power System. The simulation results showed a high accuracy using the basic algorithm under small source reactance. The maximum calculated error was − 3.9% when R_f_ = 36 Ω. When the source reactance (Xs) increased to 0.5 Ω, the error increased to 9%. This is because Xs works as a filter suppressing the fault transient and the useful wideband information. Tapped loads have only a small influence on the estimated reactance and distance of a fault imposed on the main line when Zs is very small (0.005 Ω). The magnitude of imposed error is less than 1% when the tapped load is three times the receiving end load. However, tapped loads have a significant influence when X_s_ is high and the tapped load is comparable with fault resistance. The error reached 32% when Z_tap_ = 10+j0.184 Ω (four times bigger than Zload and almost twice Z_f_ = 4.5 Ω). The proposed DEFLT with tapped load compensation worked effectively. The 32% error was reduced to 2.1% without any need for measurements from the tapped loads. The tapped load is compensated using pre-fault measurements from the line “sending” and “receiving” ends where the DEFLT transducers are installed. The simulation results were validated using experimental system tests. The estimated fault locations using the experimental system showed a very good accuracy, although it should be noted that the measurement accuracy depends very much on the accuracy of the cable impedance calibration (including frequency dependent and layout dependent non-linear effects). The DEFLT approach presented here is suitable for clearly defined compact power systems such as those present in ships, aircraft and trains, as it requires transducers and communications for the protected cables. However, the experimental results presented here confirm that it can prove very accurate fault location within these environments, and this additional task can be included with relatively low cost sensors and processing. Future work is planned to study the effects of uncontrolled rectifier loads and the influence of Renewable energy source as a second source.

## Supplementary Information

Supplementary Information.

## Data Availability

The datasets used and analyzed during the current study are available from the corresponding author on reasonable request.
