# Supplementary material for: An investigation into the robustness of a double-ended wideband impedance-based fault location technique
Source: Sci Rep. 2023 Jun 20;13:10001. doi: 10.1038/s41598-023-36541-2 (PMC10281984; doi:10.1038/s41598-023-36541-2)
Supplement: Supplementary file 1 — Supplementary Information. [file 41598_2023_36541_MOESM1_ESM.docx]

**APPENDICES**

**Appendix 1**

From Figure 5 in the manuscript, where the fault step change is the source at high non-fundamental frequencies. The following steps show the derivation process of equations (4) and (5):

*V_s_  + I_s_*Z_x1_ +* *I_x2_*Z_x2_ = V_step_* (a)

*But, I_x2_ = I_s_ + I_T_ , Then*

*V_s_  + I_s_*Z_x1_ + (I_s_ + I_T_ )*Z_x2_ = V_step_*

*V_s_  + I_s_*(Z_x1_ + Z_x2_)+ I_T_ *Z_x2_ = V_step_* (b)

*V_r_  + I_r_*Z_l-x1-x2_ = V_step_*

*V_r_  + I_r_*Z_l_ – I_r_*(Z_x1_ + Z_x2_)= V_step_* (c)

As (c) equal (b),

$V_{s}+I_{s}*(Z_{x1}+Z_{x2})+I_{T}*Z_{x2}=V_{r}+I_{r}*Z_{l}-I_{r}*(Z_{x1}+Z_{x2})$*V_s_ + I_s_*(Z_x1_+ Z_x2_)+ I_T_*Z_x2_ = V_r_ + I_r_*Z_l_ – I_r_*(Z_x1_ + Z_x2_)* $V_{s}+I_{s}*(Z_{x1}+Z_{x2})+I_{T}*Z_{x2}=V_{r}+I_{r}*Z_{l}-I_{r}*(Z_{x1}-Z_{x2})$

Rearranging for$Z_{x1}+Z_{x2}$ *Z_x1_ + Z_x2_*$Z_{x1}-Z_{x2}$, yield

$V_{s}+I_{s}*(Z_{x1}+Z_{x2})+I_{T}*Z_{x2}=V_{r}+I_{r}*Z_{l}-I_{r}*(Z_{x1}+Z_{x2})$(*I_s_ + I_r_) *(Z_x1_+ Z_x2_) = V_r_ - V_s_ + I_r_*Z_l_ - I_T_*Z_x2_*

$Z_{x} = \left( Z_{x1}+ Z_{x2} \right)= \frac{V_{r}- V_{s} + I_{r}*Z_{l}- I_{T}*Z_{x2}}{I_{s}+ I_{r}}$ (4)

To estimate the tapped branch current from the source end current, the tapped load branch is assumed to be in parallel with the source end as follows:

*V_s_ + I_s_*Z_x1_ = I_T_*(Z_T_ + Z_load1_)*  (d)

Rearranging (d), yield (4)$I_{T}= \frac{V_{s}+I_{s}*Z_{x1}}{( Z_{T}+ Z_{load1})}$
$I_{T}= \frac{V_{s} + I_{s}*Z_{x1}}{Z_{T} + Z_{load1}}$ (5)

Similarly, from Figure 6 in the manuscript, the following steps show the derivation process of equations (6) and (7):

*V_s_  + I_s_*Z_x1_ = V_step_* (e)

*V_r_  + I_r_*Z_l-x1-x2_ + I_x2_*Z_x2_ = V_step_*  (f)

But, , *I_x2_ = I_r_ + I_T_*, Then

$V_{r}+I_{r}*Z_{l-x1-x2}+(I_{r}+I_{T})*Z_{x2}=V_{step}$ *V_r_  + I_r_*Z_l-x1-x2_ + (I_r_ + I_T_)*Z_x2_ = V_step_*

*V_r_  + I_r_*Z_l_ - I_r_*Z_x1_ - I_r_ *Zx2 + I_r_* Z_x2_+ I_T_*Z_x2_ = V_step_*

*V_r_  + I_r_*Z_l_ - I_r_*Z_x1_ + I_T_*Z_x2_ = V_step_*  (g)

As (e ) equal (g),

*V_s_  + I_s_*Z_x1_ = V_r_  + I_r_*Z_l_ - I_r_*Z_x1_ + I_T_*Z_x2_*

Rearranging for *Z_x1_, yield (6)*

*(I_s_+ I_r_ )*Z_x1_ = V_r_ - V_s_ + I_r_*Z_l_ + I_T_*Z_x2_*

$Z_{x1} = \frac{V_{r}- V_{s}+I_{r}*Z_{l}+ I_{T}*Z_{x2}}{I_{s}+ I_{r}}$ (6)

$\left( I_{s}+I_{r} \right)*Z_{x1}= V_{r}-V_{s}+I_{r}*Z_{l}+I_{T}*Z_{x2}$

To estimate the tapped branch current from the Receiving end current, the tapped load branch is assumed to be in parallel with the source end as follows:

*V_r_  + I_r_*(Z_l_ + Z_x1_ + Z_x2_) = I_T_*(Z_T_ + Z_load1_)* (h)

Let $Z_{l}-Z_{x1}-Z_{x2} =Z_{x3}$ *Z_l_ + Z_x1_ + Z_x2_* = Z_x3_ and rearranging (h), yield

$I_{T}= \frac{V_{r}+I_{r}*Z_{x3}}{Z_{T}+ Z_{load1}}$ (7)

**Appendix 2**

The Table below summarised the initial parameters used in the simulated circuit of Fig. 11.

| **Parameter** | **Value** |
| --- | --- |
| Supply Voltage | 400V ph-ph |
| Supply resistance | 0.5 Ω |
| Supply Inductance | 1 mH |
| Line resistance per meter | 1.5 mΩ |
| Line Inductance per meter | 0.34 mH |
| Receiving end load Impedance | 37+j0.5 Ω |
| Tapped loads 1 impedance | 64 + j0.471 Ω |

Notes:

1- The simulation used similar inductance and resistance per each section and the inductance assumed linear with the frequency.

2- The source impedance and the tapped loads are changed as in Table 4, 6 and 7 to test the influence of source impedance and tapped load impedance on the algorithm.
